# Using machine learning algorithms and techniques for defining the impact of affective temperament types, content search and activities on the internet on the development of problematic internet use in adolescents’ population

**DOI:** 10.3389/fpubh.2024.1326178

**Published:** 2024-05-17

**Authors:** Jelena Jović, Aleksandar Ćorac, Aleksandar Stanimirović, Mina Nikolić, Marko Stojanović, Zoran Bukumirić, Dragana Ignjatović Ristić

**Affiliations:** ^1^Department of Preventive Medicine, Faculty of Medicine, University of Pristina in Kosovska Mitrovica, Kosovska Mitrovica, Serbia; ^2^Computer Science, Faculty of Electronic Engineering, University of Niš, Niš, Serbia; ^3^Department of Epidemiology, Faculty of Medicine, University of Niš, Niš, Serbia; ^4^Center for Control and Prevention of Communicable Diseases, Institute of Public Health Niš, Niš, Serbia; ^5^Institute of Medical Statistics and Informatics, Faculty of Medicine, University of Belgrade, Belgrade, Serbia; ^6^Faculty of Medical Sciences, University of Kragujevac, Kragujevac, Serbia; ^7^Psychiatric Clinic, Clinical Centre “Kragujevac”, Kragujevac, Serbia

**Keywords:** machine learning, problematic internet use, A-TEMPS-A, adolescents, internet use

## Abstract

**Background:**

By using algorithms and Machine Learning – ML techniques, the aim of this research was to determine the impact of the following factors on the development of Problematic Internet Use (PIU): sociodemographic factors, the intensity of using the Internet, different contents accessed on the Internet by adolescents, adolescents’ online activities, life habits and different affective temperament types.

**Methods:**

Sample included 2,113 adolescents. The following instruments were used: questionnaire about: socio-demographic characteristics, intensity of the Internet use, content categories and online activities on the Internet; Facebook (FB) usage and life habits; The Internet Use Disorder Scale (IUDS). Based on their scores on the scale, subjects were divided into two groups – with or without PIU; Temperament Evaluation of Memphis, Pisa, Paris, and San Diego scale for adolescents (A-TEMPS-A).

**Results:**

Various ML classification models on our data set were trained. Binary classification models were created (class-label attribute was PIU value). Models hyperparameters were optimized using grid search method and models were validated using k-fold cross-validation technique. Random forest was the model with the best overall results and the time spent on FB and the cyclothymic temperament were variables of highest importance for these model. We also applied the ML techniques Lasso and ElasticNet. The three most important variables for the development of PIU with both techniques were: cyclothymic temperament, the longer use of the Internet and the desire to use the Internet more than at present time. Group of variables having a protective effect (regarding the prevention of the development of PIU) was found with both techniques. The three most important were: achievement, search for contents related to art and culture and hyperthymic temperament. Next, 34 important variables that explain 0.76% of variance were detected using the genetic algorithms. Finally, the binary classification model (with or without PIU) with the best characteristics was trained using artificial neural network.

**Conclusion:**

Variables related to the temporal determinants of Internet usage, cyclothymic temperament, the desire for increased Internet usage, anxious and irritable temperament, on line gaming, pornography, and some variables related to FB usage consistently appear as important variables for the development of PIU.

## 1 Introduction

Technical and technological progress at the end of the 20^th^ century has lead to the wide-spread Internet use as a means of communication and a way to access desired information, which is one of the characteristics of humans as social beings. In a bigger sense and in an easier and faster way, the Internet provides individuals with a sense of belonging to a community regardless of gender, age, race, or other characteristics. In 1995, the term Internet Addiction Disorder was firstly used by Goldberg (1). Since then, almost 30 years later, controversies and criticisms of this disorder still exist and they mostly relate to the reality of its pathology. Researchers particularly point to the issue of pathologizing internet use (2). Despite many reservations, it is evident that in practice, there are many individuals seeking professional help due to losing control over Internet use, along with all the negative consequences that accompany it (3). The terms “Internet addiction” and “problematic Internet use- PIU” are used to describe a manner of Internet usage marked by excessive behavior, a lack of control, disregard for other significant activities, and persistent engagement despite experiencing negative consequences such as distress and functional impairment (4). Theoretical considerations, supported by some clinical studies (with small sample sizes), suggest that the symptoms of problematic internet use resemble those of other behavioral or psychological disorders: obsessive-compulsiveness, depression, hostility, anxiety, and emotional sensitivity (5). Based on these claims, we can conclude that the dimensions of PIU symptoms overlap with the symptoms of the disorders that most commonly lead to excessive internet use. It often happens that individuals with some problems (e.g., depression, anxiety) turn to the Internet in search of relief, which, on one hand, exacerbates their initial symptoms, and, on the other hand, intersects with new symptoms resulting from excessive internet use. This leads to a new complex of symptoms that cannot simply be equated with the initial psychological problems. The Internet is constantly evolving, and due to its capabilities, we may never be ahead of its development and fully predict all the directions in which problems and disorders related to its use can evolve (5).

Adolescents and young adults are a particularly sensitive population and they are increasingly facing very real and sometimes very serious consequences in their everyday lives arising from inadequate Internet use (6). By viewing the overall morbidity, school and student youth represent the healthiest part of the society, still, their specific characteristics make them sensitive to various negative influences coming from the living and social environment, which certainly include PIU along with all the negative risks it carries (7, 8). Besides the fact that the young people are a significant part of the population of Internet users, they also represent the largest portion of the general population with the highest risk of developing PIU (9–12). Adolescents are, also, considered the most exposed population: they are especially sensitive to the development of all types of addictions and they are the population that uses the Internet the most (13, 14). The importance of the need to protect the health of adolescents is also highlighted by the current WHO campaign titled “Global Strategy for Women’s, Children’s and Adolescents’ Health (2016–2030)” (15). There is inconsistency in the data regarding prevalence of PIU in the adolescent population. By following the analogy with other addictions, the assumption is that boys develop PIU more often. Moreover, it is expected that adolescents with better academic performance and a better socio-economic status are less likely to develop PIU.

As an “umbrella concept,” problematic Internet use encompasses a variety of problematic online behaviors (16). Until now, only problematic online gaming has entered official classifications and received its classification parameters. In the ICD-11, there is a category “disorders due to addictive behaviors,” which officially acknowledges gambling disorder and gaming disorder as the two behavioral addictions falling within this classification (17). However, research has shown that those who meet the diagnostic criteria for this addiction do indeed use the Internet for following other content and engaging in other activities (2). Let us note that there has been some genetic basis for the development PIU (18). Moreover, temperament as a biological basis of inherited personality (19) together with certain lifestyle habits that can reduce the time spent using the Internet can have a significant factor in the analysis of this complex issue. The assumption that specific activities and content on the Internet can lead to a reduced reduce but also increase risk of developing PIU is particularly interesting. So, monitoring the development of PIU through the analysis of the impact of various online activities of adolescents, the content they follow, as well as their habits in everyday life and the possible influence of temperament truly represents a challenge. Forming a nationally representative sample of adolescents was one way to address this challenge. The other way was to select analytical methods through which this kind of extensive database could be analyzed (both in terms of breadth and depth). In addition to data collection, conventional approaches to data analysis sometimes lack the capacity of variables extraction and selection. On the other hand, application of machine learning- ML methods would allow overcoming these shortcomings and enable better identification of target groups for interventions (20). ML encompasses a diverse array of methods and techniques designed to address a wide spectrum of issues. Tasks like identifying individuals with substance use disorders (21), finding patterns in neuro images, comprehending prognostic factors related to addictions and their correlations, and unraveling the genetic foundations of addiction are included. Over the past decade, various biomedical research domains, including addiction, have effectively applied machine learning in the last decade. In their review paper, Cresta Morgado et al. (22) stated that, through a PubMed database search, they discovered fewer than 200 articles pertaining to machine learning and addiction. While the number of these articles has been on the rise, articles using machine learning still constitute only 0.25% of the overall articles on addiction. Moreover, a small subset of these articles focuses specifically on behavioral addictions.

### 1.1 Machine learning techniques

Traditional data processing methods involve the use of mathematical and statistical methods to discover knowledge within a data set. They are usually based on predefined hypotheses, assumptions and mathematical models for data analysis. However, although they represent well-established methods, a problem arises when using high-dimensional and complex data sets. ML can be successfully used to solve these problems.

The ML algorithms have revolutionized the field of data processing by enabling the automatic extraction of patterns, connections and insights from complex data sets. ML can be broadly defined as computational methods which use experience to improve performance or to make accurate predictions (23). They analyze various forms of collected data. The quality and size of this data are crucial for the successful predictions made by the learner.

To successfully employ machine learning techniques for addressing various problem types, it is necessary to precisely define the problem and choose suitable ML algorithms. Different ML techniques exist based on how models are trained. Classifiers, which are classification models, predict categorical (discrete, unordered) class labels (24). The classification process unfolds in two key steps: the learning phase (creating a classification model) and the subsequent classification phase (this model is used to predict class labels for provided data points).

During the learning phase, a classification model is created by defining a predetermined set of classes or concepts (24). This constitutes the learning step during which the classification algorithm builds a model through the analysis or “learning from” a training dataset consisting of data points and their corresponding class labels. A data point, denoted as *X*, is represented by an *n*-dimensional attribute vector, *X* = (x1, x2,.., xn), describing the data point through n measurements on attributes A1, A2,..., An. Each data point, X, is assigned to a predefined class determined by a specific attribute known as the class label attribute. This attribute is represented by an unordered discrete value, where each of its possible values signifies a distinct class or category. The individual data points of the training dataset, called training data points, are randomly selected from the database.

Since the class label is known for each training data point, this step is often called supervised learning (classifier learning is supervised because each training data point is explicitly assigned to a specific class) (24). The first step of classification can be viewed as the process of learning a mapping function, *y* = *f*(*X*), capable of predicting the class label y for a provided data point *X*. In this sense, we want a mapping function that divides the data according to the class they belong to.

In the second phase, the created model is applied for classification (24). Initially, the classifier’s predictive accuracy is evaluated using a test set consisting of test data points and their corresponding class labels. These test data points are independent from the training data points and they were not utilized in the creation of the classifier. The accuracy of the classifier on the provided test dataset is calculated as the percentage of correctly classified test data points. The class label assigned to each data point is compared to the classifier’s prediction for that specific data point. If the classifier’s accuracy meets acceptable standards, it can then be employed to predict future data points where the class label is entirely unknown.

Naive Bayes is a classifier based on Bayes’ theorem. The term “naive” originates from the fact that the classifier assumes strong independence between attributes (24, 25). It is assumed that the value of a specific attribute is entirely independent of the value of any other attribute, given the assigned class label. Despite this simplifying assumption, the Naive Bayes classifier is recognized as a successful solution for practical problems related to text classification, healthcare, and similar applications.

Nearest-Neighbor classifiers use an analogy-based learning technique by comparing a given test data point with training data points that share similarities (24). Each data point, denoted as *X*, is represented as a point in an n-dimensional space. In the case of an unknown data point, the *k*-nearest-neighbor classifier explores the space to identify the *k* closest data points. These *k* training data points constitute the *k* “nearest neighbors” of the unknown data point. Closeness, or similarity, is usually defined in the context of a chosen distance metric (e.g., Euclidean distance).

Decision trees are a tree-like structure where each internal node represents a test on an attribute, each branch denotes the test result, and each leaf node (terminal node) represents a class label (24, 26). The attributes of a given data point, *X*, for which the class label is unknown, are tested using the decision tree. The path from the root node to the terminal node signifies the class prediction for the given data point.

Decision tree induction is the process of creating a decision tree based on a set of training data points with associated class labels. The induction process does not require domain knowledge or parameter tuning, making decision trees suitable for exploratory knowledge discovery (24). Decision trees generally exhibit a high level of accuracy.

Random forests, or random decision forests, are an ensemble machine learning technique that operates by constructing a multitude of decision trees during training (24). In classification, the output of the random forest is determined by the class selected by most trees.

Gradient boosting is a machine learning technique that generates a classification model in the form of an ensemble of weak classification models, typically decision trees (24). When a decision tree serves as the weak model, the resulting classification model is referred to as gradient-boosted trees.

The neural networks field was originally kindled by psychologists and neurobiologists who sought to develop and test computational analogs of neurons (27). Artificial Neural Network consists of interconnected input/output units, where each connection is assigned a weight (24). During the learning phase, the network adapts by adjusting these weights to accurately predict the class label of input tuples. Neural networks require prolonged training times, making them more suitable for applications where such durations are feasible. Determining parameters such as network topology or “structure” typically requires empirical process. Artificial neural networks offer advantages, including a high tolerance for noisy data and the capability to classify patterns on which they have not been trained. They are a valuable solution when dealing with limited knowledge of relationships between attributes and classes (24, 28).

Deep learning networks are gaining importance due to their ability to discover complex representations of raw data. Deep learning networks are composed of multiple layers of interconnected neurons that learn hierarchically more complex attributes. These models have shown impressive results in various fields, including image and object recognition, natural language processing, speech recognition, and more. The main asset of the deep learning model is the automatic extraction of the most important attributes from the data, thus achieving a more precise and finer analysis of the data.

Feature selection – “The curse of dimensionality” occurs when data sets have many attributes. High-dimensional data is a barrier to use in several fields, such as data processing, statistical analysis, and machine learning. The problems are reflected in the increased complexity of calculations, the increased demand for resources in the form of memory and processing time, and, therefore, the increased time required for training and using the model. Many attributes also increase the chance of false correlations or dependencies between attributes. As a result, models are obtained whose accuracy is lower than expected or desired, while the model itself is much more difficult to understand (29, 30).

The evolution of machine learning has led to the development of algorithms and tools that effectively reduce dimensionality due to their ability to learn complex patterns and structures within the data. These algorithms aim to learn the underlying meaning of the data and thereby discover the most informative attributes and those that most determine the difference between samples. In this way, problems caused by high-dimensional data are successfully eliminated and more efficient data analysis is promoted.

Feature selection methods have mainly three categories (29, 30):

- Filter methods – Filter methods use the general characteristics of data itself and work separately from the ML algorithm. The filter methods use the statistical correlation between a set of attributes and the target class attribute. The amount of correlation between a specific attribute and the target class attribute determines the importance of that attribute. Filter based approaches are not dependent on classifiers and are usually faster and more scalable. Also, they have low computational complexity.
- Wrapper methods – These methods use the target ML algorithm as a black box to find the best subset of attributes. Selected attributes are highly dependent on the performance of the target ML algorithm.
- Embedded methods – Feature selection occurs naturally as part of the ML algorithm. Specifically, during the classification model training process, the ML algorithm itself decides which attributes to use and which to ignore. These methods imply the dependence of the selected attributes on the specific model used during training, but it also means that no tool for external selection of attributes is necessary.

Correlation-Based Feature Selection (CFS) is a simple filter algorithm that assesses and ranks attribute subsets using a correlation-based heuristic evaluation function (31). The evaluation function selects attributes highly correlated with the class label attribute while uncorrelated among themselves. The rationale is to ignore irrelevant attributes with low correlation to the class label attribute. Additionally, redundant attributes, which are highly correlated with one or more remaining attributes, are filtered out. CFS is usually based on Pearson correlation, where all attributes are standardized for consistency.

Decision trees, including individual decision trees in different ensemble methods, can be created using different algorithms (24). Differences in algorithms for decision tree induction include how the attributes are selected in creating the tree and the mechanisms used for pruning. An attribute selection measure serves as a heuristic for determining the optimal splitting criterion that effectively separates the given training data points. The aim of these algorithms is to identify the best predictor and split point, enhancing homogeneity within each new partition (29). Therefore, if a predictor remains unused in any split, it is functionally independent of the prediction function and is excluded from the model. Ensembles of trees share this property, although certain algorithms, such as random forest, intentionally introduce splits on irrelevant predictors during the tree induction leading to an over-selection of predictors.

LASSO is primarily serving two purposes: regularization and feature selection (32). In the LASSO method, a constraint is imposed on the sum of the absolute values of the model parameters, ensuring the sum has to be less than a fixed value (upper bound). The method uses a shrinking (L1 regularization) penalizing the coefficients of the regression variables, shrinking some of them to zero. During the features selection process the variables that still have a non-zero coefficient after the shrinking process are selected to be a part of the model. The objective of this process is to minimize prediction errors. In practice, the tuning parameter *λ*, that controls the strength of the penalty, assumes important importance. When *λ* is sufficiently large. Coefficients are forced to be exactly zero, effectively reducing dimensionality. The larger the parameter *λ*, the more coefficients are undergoing shrinkage to zero. On the other hand, if *λ* = 0, an OLS (Ordinary Least Square) regression occurs.

ElasticNET (also called ELNET) regression is a statistical hybrid method that combines two of the most often used regularized linear regression techniques, LASSO, and RIDGE, to deal with multicollinearity issues when they arise between predictor variables (33). They are regularization aids in solving the overfitting issues with the models.

It is also used for regularizing and choosing the essential predictor variables that important impact the response variable (33). Ridge uses an L2 penalty, while LASSO employs an L1 penalty. Since the ElasticNET utilizes both the L2 and the L1 models, the question of choosing between either one does not arise.

A genetic algorithm (GA) is an optimization tool based on principles of evolution from population biology. To effectively identify optimal solutions, the algorithm imitates the evolutionary process of a population. It generates a candidate set of solutions, allowing them to reproduce and create new solutions. The algorithm promotes competition, providing optimal solutions with the best chance to survive solutions and populate the subsequent generation (natural selection). This iterative process enables GAs to accumulate and refine good solutions over time, improving their quality and converging to an optimization plateau.

The challenge of identifying an optimal feature subset can be treated as a complex optimization problem. Therefore, if the feature selection problem is conceptualized in terms of evolutionary biology, the GA algorithm can be applied to search for optimal feature subsets (29).

Permutation Feature Importance (34) is an approach that measures the impact on the prediction error when the values of a feature are permuted, disrupting the relationship between the feature and the actual outcome. A feature is considered as “important” if shuffling its values increases the model error, indicating the model’s reliance on that feature for prediction. On the opposite side, a feature is considered “unimportant” if shuffling its values leaves the model error unchanged, signifying the model’s disregard for that feature in prediction. This model is very popular for ML models that are commonly labeled as “black-box” models, such as artificial neural networks. Nonetheless, there are instances where neural network models can provide explanatory insights using the permutation feature importance approach. The permutation feature importance metric was initially introduced for random forests in (35, 36) and subsequently expanded with a model-agnostic version in (37, 38).

By using algorithms and ML techniques, the aim of this research was to determine the impact of the following factors on the development of PIU: sociodemographic factors, the intensity of using the Internet, different contents accessed on the Internet by adolescents, adolescents’ online activities, life habits and different affective temperament types.

## 2 Methods

### 2.1 Sample

High school students, aged 16 and 17, were included in the research. The research was conducted as an observational cross-sectional study. In selecting schools, in which the research was conducted, the representation of the school was respected according to the official regionalization of the Republic of Serbia, which includes five regions (39). At the time when the research was created in the Republic of Serbia, 462 high schools existed, based on the data from the Ministry of Education and Science of the Republic of Serbia (40). The sample was stratified first by regions, then by cities within regions and finally by schools within cities. In schools, based on the number of classes, a random sample determined which departments would participate. The final stratified proportional sample included 48 high schools (about 10% of all schools). The study was conducted within a time frame of 45 days.

In accordance with the Law on the patients’ rights, each participant was informed about the research. In the results of the survey, questionnaires filled in by the participants who gave their written consent for the participation in the research were included, in accordance with Article 25, paragraph 6 of the stated law. The questionnaires were filled out during school classes.

### 2.2 Instruments

The following questionnaires were used in the research:

#### 2.2.1 A four-part questionnaire

A four-part questionnaire was created for this research, within which data was obtained about:

- The socio-demographic characteristics of the participants (gender, age, satisfaction with socio-economic status, academic achievement).
- The intensity of the Internet use: Internet Use (in years); Internet Use (hours per week); Internet Use (hours per day); Internet Use (on holiday) – The given question was: Do you use the Internet more while on holiday? Attitude about the time on the Internet –the given question was: If it were possible, would he/she spend more time on the Internet?
- In the third part, subjects answered questions about the searched content (Politics, Business, Sports, Computers and technology, Arts and culture, Education, Pop culture, Pornography, Music, Travel/Tourism, Health and medicine, Science, Religion) and types of activities (Communication by e-mail, Social networks, Communication on the forum, Communication on the blog, Targeted Internet search, Surfing, Expert Advice, Search for favorite websites, Reading the news, Online games, Reading and downloading books and texts, Downloading music and movies, Internet for school, Online courses) on the Internet. A five-level Likert scale (from 1 “never “to 5 “frequently“) was given for every online content category and online activity category. Also we asked participants about the Facebook use (Everyday FB use, Average time spent on FB, FB use for gaming, FB use for– chatting, FB use for– visiting groups, FB use for- reading posts, FB use for- publishing statuses, FB use for- sharing music, photos etc.).
- In the fourth part, subjects answered questions about habits (playing sport, eating fast food, drinking alcohol, drinking energetic drinks, coffee consumption and smoking).

#### 2.2.2 Internet use disorder scale – IUDS

The instrument was standardized and validated into the Serbian language (41). The scale consists of 18 items which participants rate according to a five-level Likert’s scale (from 1 “minimally” to 5 “completely”), including the questions connected with the compulsive Internet use, the symptoms of abstinence and increased tolerance, as well as the questions linked with the problems at work and school (Cronbach’s alpha coefficient *α* = 0.815). According to subjects’ scores on the scale (cut-off 39/40), they were divided into two groups (with or without PIU).

#### 2.2.3 Temperament evaluation of Memphis, Pisa, Paris, and San Diego-autoquestionnaire (TEMPS-A) for adolescents – A- TEMPS-A

TEMPS-A is a self-evaluation questionnaire which determines the belonging to one of the following affective temperaments: depressive, cyclothymic, hyperthymic, irritable and anxious. According to the definition of affective temperament, hyperthymic temperament is released from depressive characteristics, and vice versa, depressive temperament does not contain any of hyperthymic components. Cyclothymic and irritable temperaments are the successive and simultaneous mixture of hyperthymic and depressive characteristics, while anxious are closely connected with depressive temperament (42–44). The current version of the questionnaire has been developed over the last 10 years. The questionnaire is standardized in psychometric studies firstly in six languages and in six cultures: American English (Memphis and San Diego), Italian, French, German (versions from Minster and Hall), as well as, Turkish and Japanese. The factorial structure is consistent in all the mentioned cultures, and the scale has a high internal consistency. The scale is validated also in Serbian (45). A- TEMPS-A is a completely new version of the scale adjusted to the adolescents’ age [Cronbach’s alpha coefficient α = 0.77, and the average test–retest coefficient (rho = 0.84)] (46).

#### 2.2.4 Statistical analysis

Primarily obtained data were analyzed using descriptive statistical methods. Measures of central tendency (arithmetic mean), measures of variability (standard deviation) and indicators of structure expressed as percentages were used as descriptive statistical methods. For these analyses, the data were processed using the IBM SPSS Statistics 22 software package – SPSS Inc., Chicago, IL, United States.

##### 2.2.4.1 ML algorithms and ML techniques

For the purpose of this paper, authors used different machine learning models and techniques implemented as a part of the of the Python scikit-learn ML framework (version 1.2.2) (38). The ML pipeline used in this paper was implemented as a Python (version 3.8) (47) script using Jupyter notebook tool (48). The implemented pipeline consists of the following steps:

- Data set preprocessing.
- Attribute selection using filter methods.
- Training binary classification models (for data points labeled with PIU present or not) using cross validation (24) to assess and compare results of different models.
- Optimization of classification models hyperparameters.
- Attribute selection using embedded methods.
- Attribute selection using wrapper methods.

These ML pipeline steps will be explained in detail later when discussing machine learning results. The results are presented in both tabular and graphical form.

## 3 Results

### 3.1 Socio – demographic characteristics of participants

The final sample included 2,113 adolescents, out of 2,239 initially surveyed and out of which 56% were female and 44% male, with a mean age of 16.73. Detailed data concerning socio-demographic characteristics of the sample is shown in Table 1.

**Table 1:** Characteristics of participants according to academic achievement and economic status.

Characteristics	*n*	%
Academic achievement		
Best	806	38.1
Very good	782	37.2
Good	475	22.6
Passable	40	2.0
Insufficient	–	
Economic status		
Low	105	5.0
Lower middle	223	10.7
Middle	830	39.7
Upper middle	574	27.5
High	357	17.1

### 3.2 The intensity of internet use

The biggest percent of adolescents in Serbia weekly spends over 20 h on the Internet.

According to the obtained data, the maximum time spent in continuity in 62.8% of participants was 5 h. In total, about a fourth of participants spent 5–10 h on the Internet in continuity, and 4.5% of participants spent over 20 h in continuity. It needs to be noted that this was not the case with every day continual Internet use (Table 2).

**Table 2:** The Intensity of Internet use.

Characteristics	Mean ± SD
Number of years of internet usage	5.54 ± 2.27
Weekly use in hours	n (%)
Up to 2 h	216 (10.4)
2–5 h	363 (17.2)
5–10 h	396 (19.0)
10–15 h	354 (17.0)
15–20 h	304 (14.6)
Over 20 h	446 (21.4)
Maximum time spent on the Internet in continuity daily	*n* (%)
Up to 5 h	1281 (62.8)
5–10 h	454 (22.2)
10–15 h	127 (6.2)
15–20 h	79 (3.9)
Over 20 h	99 (4.5)
Time spent on the Internet in the period of studying in relation to holidays	*n* (%)
More	638 (30.5)
Less	428 (20.5)
The same	1022 (49.0)
Attitude about time on the Internet	*n* (%)
If they could, they would spend more time	315 (15.1)
If they could, they would spend less time	1771 (84.9)

### 3.3 Interests and behavior of participants on the internet and FB

The participants in this study more often search the Internet for contents related to music, followed by online content categories regarding education, sports and health and medicine (Table 3).

**Table 3:** Self reported frequency of searching for content on Internet (on the scale between 1–5).

Online content categories	Whole sample
	Mean ± SD
Politics	1.65 ± 0.94
Business	1.68 ± 0.99
Sports	3.23 ± 1.38
Computers and technology	2.46 ± 1.28
Arts and culture	2.73 ± 1.25
Education	3.27 ± 1.17
Popular culture	2.85 ± 1.33
Pornography	2.28 ± 1.60
Music	4.82 ± 0.57
Travel/Tourism	2.94 ± 1.22
Health and medicine	3.04 ± 1.21
Science	2.92 ± 1.24
Religious content	2.0 ± 1.14

Our participants’ most frequent online activity is Social networks, targeted search for information and, after that, downloading music and movies (Table 4).

**Table 4:** Self-evaluation of Internet activities (on the scale between 1 and 5).

Online activity categories	Whole sample
	Mean ± SD
Communication by e-mail	2.73 ± 1.37
Social networks	4.45 ± 0.96
Communication on a forum	1.73 ± 1.02
Communication on a blog	2.56 ± 1.30
Targeted search for information	4.31 ± 0.88
Network search – surf	3.12 ± 1.32
Expert advice	1.57 ± 0.93
Search for favorite websites	3.95 ± 1.17
Reading the news	3.35 ± 1.27
Online games	2.44 ± 1.49
Reading and downloading books and texts	2.81 ± 1.36
Downloading music and movies	4.25 ± 1.06
Internet for school	3.72 ± 1.17
Online courses	1.68 ± 1.03

When it comes to activities on FB, a detailed results was given in Table 5. Every day, FB is used by 83.3% of participants. On average, participants spend 4.09 ± 4.92 h on FB. The most frequent participants’ activity on FB is chatting.

**Table 5:** Interests and behavior of participants in relation to FB.

Characteristics	Whole sample
Uses FB everyday B	*n* (%)
Yes	1735 (83.3)
No	273 (13.1)
Does not have FB	76 (3.6)
Average time spent on FB, mean ± SD	4.09 ± 4.92
Activities on FB	*n* (%)
Reads posts on FB	1040 (49.2)
Writes FB statuses	331 (15.7)
Shares content on FB	720 (34.1)
Plays games on FB	272 (12.9)
Chats on FB	1459 (69.0)
Visits groups on FB	476 (22.5)

### 3.4 Characteristics of participants according to affective temperaments and internet use disorder scale – IUDS

The correlation among temperaments was fluctuating from weak to medium (Table 6). The strongest positive correlation was among depressive, cyclothymic, and anxious temperaments. Hyperthymic temperament was in negative correlation with all other temperaments. The prevalence of PIU in our sample was 28%.

**Table 6:** Correlation among affective temperaments.

	Cyclothymic	Hyperthymic	Irritable	Anxious
Depressive	0.399^**^	−0.379^**^	0.307^**^	0.427^**^
Cyclothymic		−0.147^**^	0.381^**^	0.395^**^
Hyperthymic	−0.147^**^		−0.025	−0.198^**^
Irritable	0.381^**^	−0.025		0.259^**^
Anxious	0.326^**^	−0.202^**^	0.219^**^	

^**^p < 0.01.

### 3.5 Characteristics of participants according to selected lifestyle habits

Our participants, on average, play sports 4 days in a week (3.94 ± 1.99), with the average duration of training a bit longer than 1 h (64.91 ± 41.36). A bit less than a half of participants consume energy drinks every day (43.6%) and that, on average, 136 mL. The great majority of participants (81.1%) eat fast food every day. A bit less than a third of participants are smokers-in total, 494 of them (23.6%). Coffee is also consumed by about a third of participants, more precisely, 661 (31.8%) participants. Alcoholic drinks are consumed by 482 participants, which makes up for 23.0% of the sample.

### 3.6 Machine learning results

Before creating different machine learning models, the original data set was prepared using different techniques for data cleansing and pre-processing. After that, data points that had a missing value for the class-label attribute were deleted from the data set. Missing values for other attributes were handled using most frequent value or mean value depending on the nature of the attribute using KNNImputer implementation from the scikit-learn framework (38). During the pre-processing step, data outliers were detected using the isolation forest approach (49) implementation from the scikit-learn framework. Detected outliers were removed from the data set. At the end of this step, the values of all attributes are normalized and standardized using RobustScaler, MinMaxScaler, and StandardScaler implementations from scikit-learn framework (38).

**Figure 1:**
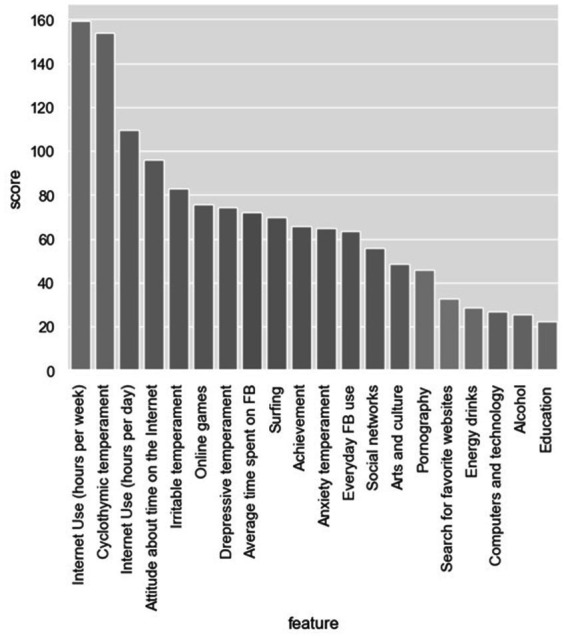
Feature selection using Pearson’s coefficient.

The next step was attribute selection using filter methods. As already mentioned, these methods are independent from ML models and can be applied separately. CFS calculates the Pearson correlation between each attribute and the class-label attribute, selecting only those attributes that have a moderate-to-high positive or negative correlation (close to −1 or 1) and drops those features with a low correlation (a value close to zero) (Figure 1). As seen in Graph 1, time spent online – Internet use (hours per week) is of the highest importance. It is followed by cyclothymic temperament, Internet use (hours per day), then attitude about time on the Internet and, immediately after, irritable temperament and online gaming.

In the third step, various ML classification models like decision tree, Naïve Bayes, *K*-Nearest Neighbors, Random forests and Gradient boosting on our data set were trained. Binary classification models were created (class-label attribute was PIU value). The dataset was divided into training and test sets in a 90:10 ratio (stratified split with a shuffle). Hyperparameter optimization was performed for all models using the grid search with cross-validation method (24), and the validation of the obtained models was performed using *k*-fold cross-validation with the value of *k* set to 10 and stratified shuffle splits.

**Figure 2:**
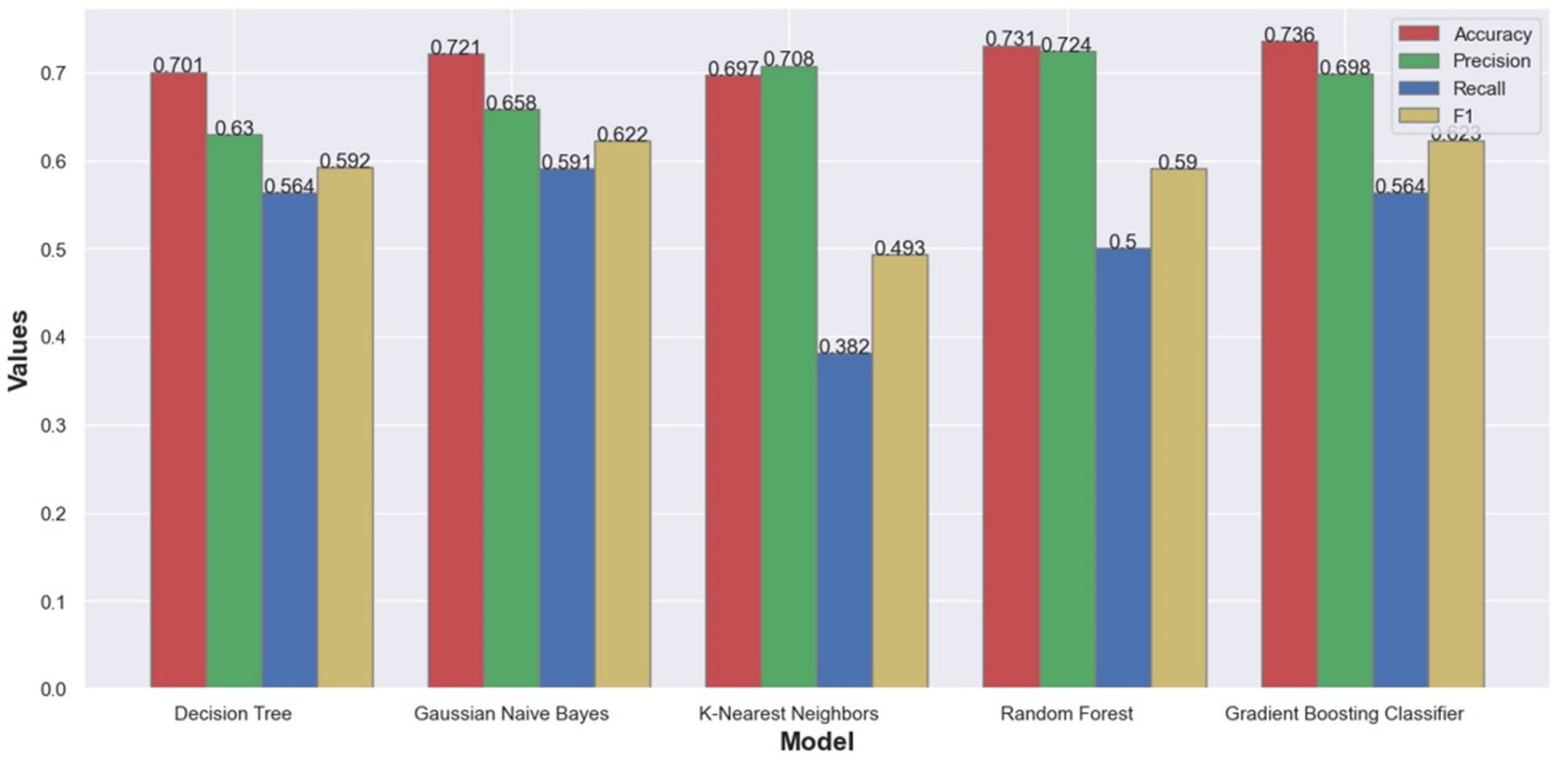
Bar Chart with classification metrics on our dataset from different machine learning algorithms.

Trained models were compared using standard classification metrics: accuracy, precision, recall, and F1-measure. Different classification metrics were used to analyze the quality of the trained models (24). Accuracy is the most common classification metric and it measures how often the classifier models correctly predicts results. The accuracy can be defined as the ratio of the number of correct predictions and the total number of predictions. Accuracy metric can be misleading in situations where the dataset is not well balanced regarding class-label attributes. In such scenarios additional metrics are usually employed. Precision explains how many of the correctly predicted cases actually turned out to be positive and this metric is useful in the cases where false positive predictions are a higher concern than false negative predictions. Recall (or sensitivity) metric explains how many of the actual positive cases we were able to predict correctly with our model. Recall is a useful metric in cases where false negative predictions are of higher concern than false positive predictions. F1-measure is the harmonic mean of precision and recall and gives a combined idea about precision and recall metrics. Because the used dataset is not well balanced, we have more data points with PIU value of zero, we cannot rely only on accuracy. For that reason, additional metrics were computed and from Figure 2 we can see that Random forest was the model with the best overall results.

**Figure 3:**
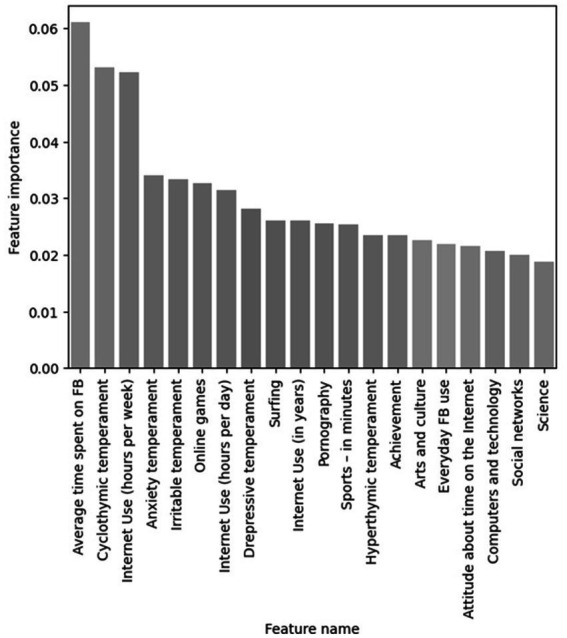
Feature selection – random forest.

After creating models, the first embedded feature selection approach using Random forest can be applied (Figure 3). Measures which are used to evaluate classifier algorithms show that the accuracy of a classifier is acceptable and that the percentage of correctly classified positive tuples is 74, while the percentage of positive tuples that are correctly classified in relation to the total number of positive tuples is 77 and 68% (Table 7). Because of this, it can be concluded that the classifier accuracy is high and that its quality is acceptable.

**Table 7:** Evaluation of classifiers – random forest.

Accuracy for the model with all features:0.74				
Classification report for the model with all features:				
	Precision	Recall	f1-score	Support
0.0	0.77	0.85	0.81	242
1.0	0.68	0.56	0.61	140
				
Accuracy			0.74	382
Macro avg.	0.73	0.70	0.71	382
Weighted avg.	0.74	0.74	0.74	382

If the graph showing the most important features selection for random forest are viewed, it can be seen that the variable of highest importance is Average time spent on FB, then Cyclothymic temperament, Internet use (hours per week) but also Anxiety and Irritable temperament. They are closely followed by Online games.

**Figure 4:**
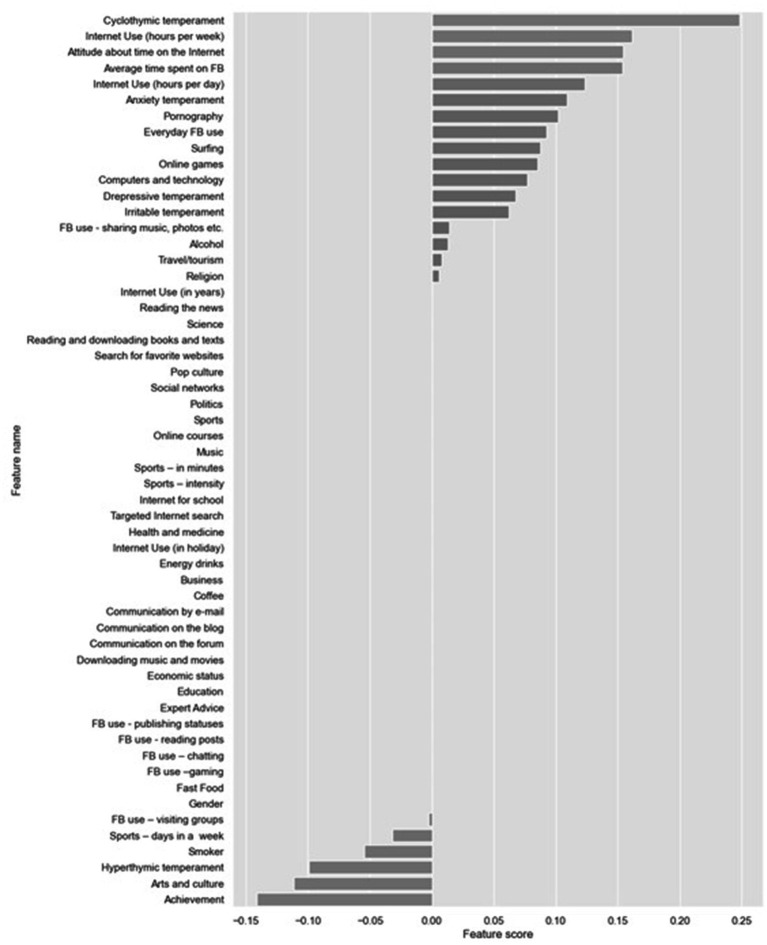
Feature selection using LassoCV.

**Figure 5:**
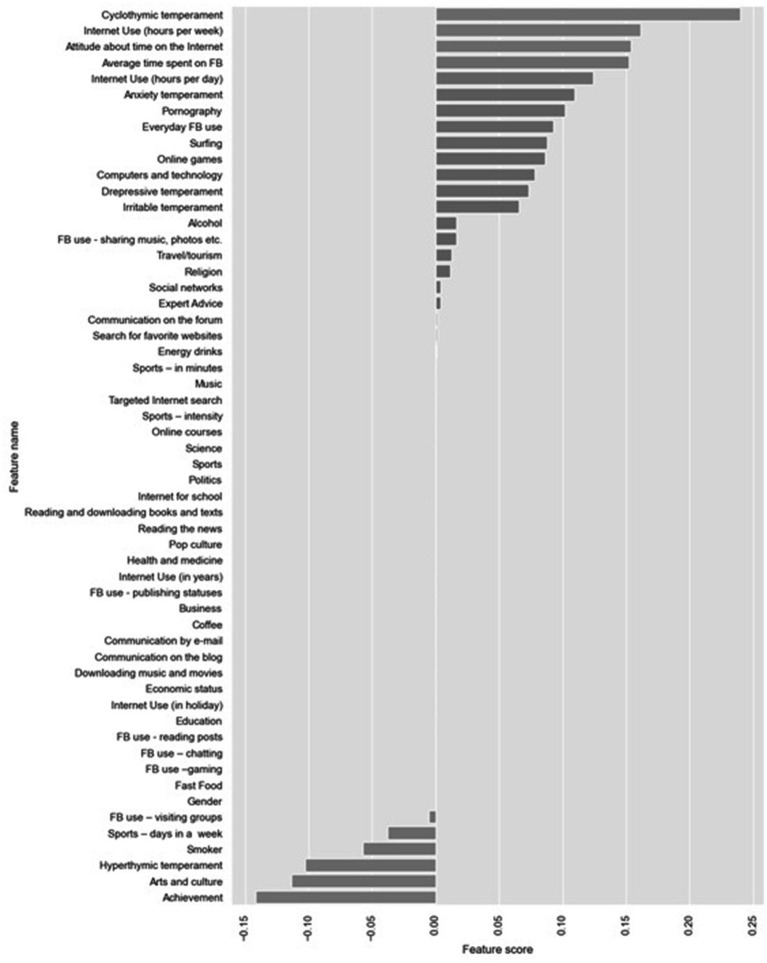
Feature selection using ElasticNetCV.

Next, Lasso and ElasticNet embedded feature selection methods were applied (Figures 4, 5).

On Figure 4, the feature selection for the most important feature using Lasso techniques can be seen. Variables which are most important for the development of PIU can be seen. It is especially important to note that a group of variables having a protective effect (regarding the prevention of the development of PIU) was found with these techniques.

In the first group, the variables are: Cyclothymic temperament, Internet use (hours per week), Attitude about the time on the Internet, Average time spent on FB, Internet use (hours per day) but also aimless Internet browsing, i.e., Surfing as well as Pornography.

Variables with protective effect are: Sports, Smoker, Hyperthymic temperament, Arts and culture, and Academic Achievement. On Figure 5, the feature selection for the most important feature can be seen by using ElasticNetCv. It can be observed that both techniques have led to the selection of identical variables of importance.

In the final step of the created ML pipeline, genetic algorithm wrapper feature selection was applied. Thirty-four important variables that explain 0.76% of variance were detected using the genetic algorithms. The variables of importance are: Arts and culture, Average time spent on FB, Business, Coffee, Communication by e-mail, Communication on the blog, Communication on the forum, Cyclothymic temperament, Downloading music and movies, Depressive temperament, Energy drinks, Everyday FB use, Expert Advice, FB use – reading posts, Fast food, Gender, Health and medicine, Hyperthymic temperament, Internet use (hours per day) Internet use (hours per week), Internet use (on holiday), Irritable temperament, Online games, Politics, Pop culture, Pornography, Reading and downloading books and texts, Religion, Science, Search for favorite websites, Sports – days in a week, Surfing, Travel/tourism (Table 8).

**Table 8:** Genetic algorithm feature selection.

Feature importance	
Achievement	False
Alcohol	False
Anxiety temperament	False
Arts and culture	True
Attitude about time on the Internet	False
Average time spent on FB	True
Business	True
Coffee	True
Communication by e-mail	True
Communication on a blog	True
Communication on a forum	True
Computers and technology	False
Cyclothymic temperament	True
Downloading music and movies	True
Depressive temperament	True
Economic status	False
Education	False
Energy drinks	True
Everyday FB use	True
Expert Advice	True
FB use – publishing statuses	False
FB use – reading posts	True
FB use – sharing music, photos etc.	False
FB use – chatting	False
FB use – visiting groups	False
FB use –gaming	False
Fast Food	True
Gender	True
Health and medicine	True
Hyperthymic temperament	True
Internet use (hours per day)	True
Internet use (hours per week)	True
Internet use (on holiday)	True
Internet use (in years)	False
Internet for school	False
Irritable temperament	True
Music	False
Online courses	False
Online games	True
Politics	True
Pop culture	True
Pornography	True
Reading and downloading books and texts	True
Reading the news	False
Religion	True
Science	True
Search for favorite websites	True
Smoker	False
Social networks	False
Sports	False
Sports – days in a week	True
Sports – in minutes	False
Sports – intensity	False
Surfing	True
Targeted Internet search	True
Travel/tourism	True

**Figure 6:**
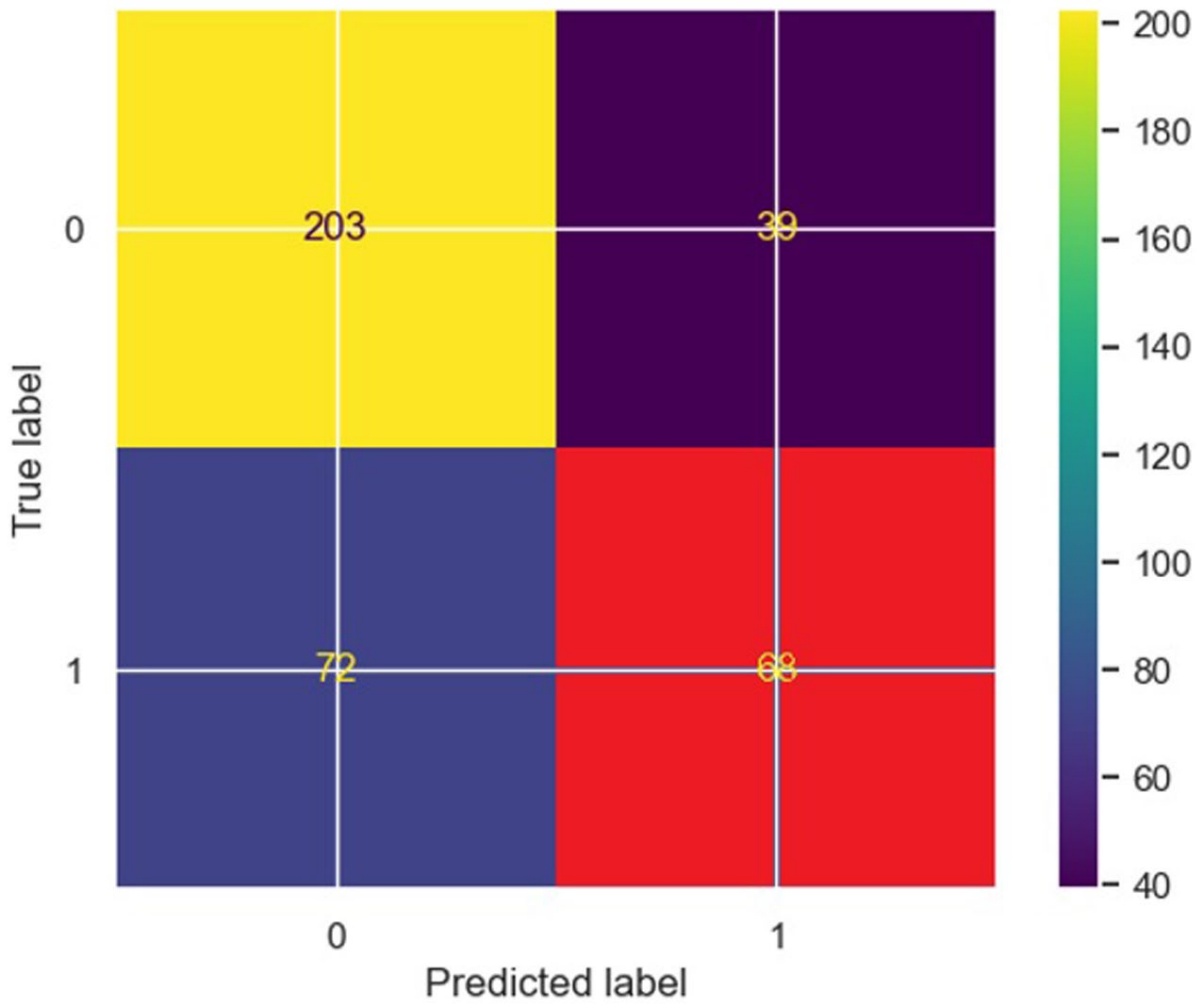
Neural network confusion matrix.

Finally, an artificial neural network model for binary classification (class-label attribute was PIU value) was created using our data set. This model performs best among all classification models trained using our data set (Figure 6). Classification metrics for the artificial neural network model were: accuracy 0.80, precision 0.82/0.78, recall 0.77/0.83, and F1 0.80/0.80.

A permutation feature importance method was applied on a trained ANN classification model in order to obtain feature importance measures. ELI5 implementation of permutation feature importance method was used (Table 9). As can be seen, variables of importance are Internet use (hours per week), FB use – reading posts, Sports, Pornography, Internet use (hours per day), Alcohol, Irritable temperament, Achievement, Coffee.

**Table 9:** Feature importance neural network.

Internet use (hours per week)	0.0541 ± 0.0089
FB use – reading posts	0.0426 ± 0.0060
Sports	0.0406 ± 0.0115
Pornography	0.0371 ± 0.0099
Internet use (hours per day)	0.0362 ± 0.0074
Alcohol	0.0320 ± 0.0055
Irritable temperament	0.0304 ± 0.0060
Achievement	0.0292 ± 0.0076
Coffee	0.0283 ± 0.0084
FB use – visiting groups	0.0267 ± 0.0086
Smoker	0.0267 ± 0.0087
Online games	0.0266 ± 0.0067
Arts and culture	0.0261 ± 0.0074
Communication on the blog	0.0261 ± 0.0061
Cyclothymic temperament	0.0257 ± 0.0078
Sports – days in a week	0.0257 ± 0.0061
FB use – chatting	0.0252 ± 0.0056
Fast food	0.0239 ± 0.0094
Computers and technology	0.0233 ± 0.0086
Attitude about time on the Internet	0.0223 ± 0.0055

## 4 Discussion

By applying a ML model, we tried to contribute to a better understanding of the multifactorial nature of PIU. ML has been applied for enabling data analysis to be done in the best possible way and, thereby, in order to overcome eventual deficiencies that standard statistical methods may lead to. Namely, ML determines various models and when a model that yields the best results is obtained, an analysis of features (variables) for that model follows. Their importance is examined and, in this way, it can be concluded what the real-world impact of these variables is, or in this case, the effect on the development of PIU of the analyzed variables.

By applying the IUDS scale, it was determined which participants have PIU and which do not- while the score on the scale that classifies the participants into one of the mentioned categories became the class attribute. The classification‘s role is to select a model that describes the class attribute as function of the values of the other attributes. While, classifier evaluation actually estimates the model, i.e., to what extent what is determined by the model corresponds to actual classification of examples. Each of the obtained models, through the analysis of the obtained most important variables (features) for that model, will enable us to understand the factors that affect the development of risky adolescent Internet behavior which ultimately leads to PIU.

Assessing the prevalence of PIU is challenging due to the initial absence of consensus diagnostic criteria. In studies employing a cut-off at an Internet Addiction Test (IAT) score of ≥50, the prevalence ranged between 8 and 27.2% (50–58). This huge variability in prevalence rates may be due to the overall poor quality of epidemiological studies in this field (59) related to the constant evolution of the technology itself, the screening instruments used, the target population and the lack of consensus about the establishment of cut-off points or the criteria used to define a “disorder” (60–62). The prevalence of PIU in our sample was 28%. Nogueira-López et al. (63) introduced a new approach to determining the prevalence of PIU. It is important to stress that different prevalence were obtained, depending on the framework which had been used. According to DSM-5 framework, they found a prevalence of 33% for problematic Internet use and 3.1% for problematic gaming. However, when a more conservative approach was implemented, there was a decrease in prevalence rates – 2.98% for problematic Internet use and 1.8% for problematic gaming.

We believe that this analysis contributes to a better and clearer understanding of the frequency of PIU and opens up new possibilities for further research. However, we must note that in our study, we used the only scale that measured PIU and was validated in the Serbian language.

Through the analysis of variables highlighted by various models, it becomes evident that time-related factors, including daily and weekly internet usage, are important for understanding development of Problematic Internet Use (PIU). From the moment when researches about the Internet started, time determinant has been examined in the context of their influence on the PIU development. They are not a certain indicator of addictive behavior, but they have proved to be very reliable, except in the cases when the Internet is used for professional purposes or for school obligations. Although today we know that contents are also essentially important, the time spent on the Internet impacts a lot on development the PIU, especially in the adolescents’ group.

When employing a feature selection approach, such as Random Forest, in addition to the previously mentioned time-related variables, important factors for PIU development include cyclothymic, anxious, and irritable temperaments, as well as online gaming. Notably, it’s interesting to analyze the importance of time spent on Facebook as a variable of utmost importance at this point. The first which draws the attention in our results is a fact that the time spent on the Internet and time spent on FB do not overlap. Maybe the reason for that is because our participants see FB totally separated from the Internet, and they do not experience it as a part of the Internet. It surely shows that adolescents compared to adults differently perceive the Internet and contents on it.

Let us now analyze the potential influence of different types of affective temperaments on the development of PIU. For now, based on our information, there is only one research with the model similar to ours, and in it anxious affective temperament was in the greatest correlation with the PIU (64). The common neurobiological base for the development of behavioral addictions and substance addiction is today explained very well. Following this parallel, we may find answers to the question of the influence of temperament on the development of PIU.

Namely, in a large number of study, cyclothymic temperament is always in the strongest relationship with substance addiction (65). The assumption is that in the foundation of cyclotomy is a biological mechanism which leads to the emotional instability and which makes people with predominant cyclothymic temperament in the greatest risk for addiction development. In short, the cyclothymic temperament might impact the development of drug dependence by facilitating the initial engagement with the substance at different stages, driven by an unregulated pursuit of pleasure. Later, this temperament may contribute to the progression of the addiction by enhancing the euphoric effects and gratification experienced in response to the substance (66, 67). Our results also have indicated the influence of anxious and irritable temperament on the development of PIU According to some studies, irritable temperament is the next in the correlation with addictions but based on some others it is anxious (68). Although there are above - mentioned parallels between these two types of addiction, our assumption is that anxious temperament almost certainly singled out as a special predictive factor for PIU because of the one Internet specificity. The Internet use gives adolescents a certain feeling of freedom in expression and communication. Therefore, we can assume that anxious people especially suits to be on the Internet, because it excludes exactly that what is a problem to them, a social contact face to face. Again, this is just on the level of assumption founded on the well-known clinical characteristics of the anxiety disorder, with the fence that anxious temperament can only represent the foundation for its development.

As we have already said, online gaming is today recognized as a special type of behavioral addiction, so the importance of its component in the development of PIU disorder is not surprising. Gaming disorder is fundamentally defined by a presence of a persistent and dysregulated pattern of engagement that is linked to adverse consequences in social, occupational, familial, and educational aspects, as well as functional impairment (69, 70).

The most important features using Lasso techniques were divided into two groups: variables s important for the development of Internet Use Disorder (IUD) and variables with a protective effect regarding the prevention of PIU.

In the first group of variables, among those we have described, other important variables include: Attitude about the time on the Internet and Pornography. Variable Attitude about the time on the Internet is actually the answer to this question: “If you could, would you spend more time on the internet?” Those who answered positively have a higher risk of developing PIU. This result is expected because one of the characteristics of addiction is the increase of tolerance (71).

As for the Pornography variable in our research, the question was only aimed at pornographic contents, which is the most popular type of activities from this group of contents (72–74). Apart from the fact that pornographic contents are easily available, they enable adolescents to be totally anonymous on one hand and to be engaged in sexual experiences on the other hand, without those risks which these experiences exists in everyday life (75). However, although pornographic and other contents of sexual connotation are most frequently researched from the aspect of the possible impact on the development of the PIU, they are not always harmful (e.g., social personal) (76, 77). On the other hand, for the important percentage of adolescents in researches, they clearly show a seriously bad impact on their life (78–80).

However, a much more interesting group of variables is those with a protective effect. Lasso and ElasticNet features selection methods are recognized same variables: Sport, Cigarettes, Hyperthymic temperament, Arts and culture and Academic Achievement. It is interesting that, except in two studies (81, 82), according to our information, it is not monitored what on the Internet might recommend to adolescents to work or follow, which would be protective for them. Such a concept would even be a foundation for a preventively protective model, which would be further developed. Adolescents who engage in sports more frequently during the week are somehow protected from the development of PIU. Playing sport, they decrease the time spent on the Internet and have healthier habits. A series of studies speak about the protective role of sport compared with the development of mental disorders, and addiction, as well. Also, it is known that playing sport is frequently included in some of the therapeutic programs in rehabilitation centers.

Hyperthymic factor clearly stands as a protective factor. In many earlier types of research which results are united in a meta-analysis of the TEMPS –A scale use (83), hyperthymic temperament clearly differs on one hand and others affective temperaments on the other hand (65). Characteristics of the mere temperament which is active, hilarious, full of life, but also responsible, with the strongly developed sense for leadership do not go with any psychopathology form. What is completely new in our paper is that A-TEMPS-A scale showed as good for identification of this temperament in the adolescent population. Regarding academic achievement, based on earlier research (82, 84–86), we know that family economic disadvantage and poorer academic performance are predictors of PIU. What makes this result particularly noteworthy is that through complex machine learning methods, good academic achievement has been singled out as a variable with strong protective effect (regarding the prevention of the development of PIU).

An artificial neural network model for binary classification has identified “FB use – reading posts” as one of the most important variables. In addition to this, two more variables not previously mentioned in the analysis have been recognized in this model: Alcohol and Coffee. The assumption is that the connection between alcohol and coffee consumption and PIU might be attributed to the fact that bad habits often co-occur. However, it’s important to mention that our question was only related to whether our respondents consume coffee and alcohol. We did not ask in detail when and how much they consume it. We cannot know if our participants, for example, consumed them while they were using the Internet. Nevertheless, this is certainly a result worthy of attention. Finally, let us analyze the variable related to Facebook usage. Let us briefly touch on Facebook as the only social network we analyzed in our research. We did not have a structured questionnaire, but the questions were quite detailed. It’s not surprising that in several models, some aspects of using this social network emerged as strong predictors of PIU. Nowadays, Facebook (FB) is an inseparable component of the adolescent ‘social lives. Among other functions, it contributes to improved information exchange of diverse content and serves as a platform for communication. Nevertheless, recent studies indicate that the prominent social network may pose as an emerging mental health concern (87). Neurobiological processes such as sensory associative learning and emotions, that underlie adaptive responses to environmental stimuli have evolved over time in an environment distinct from the current one. The rise of the social media introduces a constant interaction with new types of stimuli and rewards associated with social media. This exposure may trigger maladaptive social-media–related decision-making and behaviors. Therefore, it is unsurprising that the growing global prevalence of social media use has been accompanied by rising concerns regarding its impact on psychobiological processes and problematic use of social media (88–90).

Our study has certain limitations. Firstly, it was conducted before the COVID-19 pandemic. It is known that Internet usage in all age groups has changed (91). Meng et al. (92) specify a higher prevalence of different forms of addiction associated with PIU (smartphone addiction, social media addiction, cybersex addiction) in their meta-analysis. Furthermore, the results of the research conducted in Serbia indicate that there is a potential correlation between the changes in Internet usage during the COVID-19 pandemics and the symptoms of depression, anxiety, and stress (93).

Also, although the assumption is that the majority of our respondents are healthy, it is certain that this was not the case for all of them. For further research, a similar study should include a psychiatric interview as part of the methodology. Additionally, there are new scales that more precisely measure what we tracked in this study. Their application in some future research, along with the mentioned psychiatric interview, would certainly contribute to a higher quality and clearer understanding of complex PIU.

In conclusion, we can say that variables related to the temporal determinants of Internet usage, then cyclothymic temperament, the desire for increased Internet usage, irritable temperament, on line gaming, pornography, and some variables related to Facebook usage consistently appear as important variables for the development of PIU. This conclusion is in some way in line with the predictions made by Fineberg et al. (16) in their recent overview of PIU in Europe. They emphasize that, considering the strength of current scientific evidence and striking a balance between avoiding the over-pathologization of everyday behaviors and recognizing conditions of clinical importance that warrant public health attention, behaviors identified as most likely to qualify for diagnosis within this category include problematic online pornography viewing, shopping/buying and potentially social media use (16). It is also worth noting that academic achievement and engagement in sports consistently emerge in three models as variables with a protective factor. At the end, let us mention that as medical doctors working with adolescents (both in clinical practice and in the development and implementation of preventive programs), when forming this research, we had the idea of obtaining several factors that can be reasonably associated with PIU. When we look at our results, we can assume that we have to some extent succeeded in doing so.

## Data availability statement

The datasets presented in this article are not readily available because our participants were 16 and 17 years old. Requests to access the datasets should be directed to jelena.jovic@med.pr.ac.rs.

## Ethics statement

The study obtained the approval of the Ethical Committee of the Faculty of Medicine, University of Pristina in Kosovska Mitrovica, Serbia. The studies were conducted in accordance with the local legislation and institutional requirements. Written informed consent for participation in this study was provided by the participants’ legal guardians/next of kin.

## Author contributions

JJ: Conceptualization, Funding acquisition, Methodology, Writing – original draft, Writing – review & editing. AĆ: Methodology, Writing – review & editing. AS: Conceptualization, Formal analysis, Writing – review & editing. MN: Formal analysis, Writing – review & editing. MS: Funding acquisition, Writing – review & editing. ZB: Formal analysis, Writing – review & editing. DI: Methodology, Writing – review & editing.
